# The Environmental Pathways and Veterinary Health Implications of Microplastics and Nanoplastics: A Comprehensive Evaluation of Emerging Contaminants from a One Health Perspective

**DOI:** 10.3390/vetsci13020202

**Published:** 2026-02-20

**Authors:** Muhammad Farhan Rahim, Saisai Gong, Kewei Li, Chuxian Quan, Farah Ijaz, Yan Li, Quan Mo, Jiakui Li

**Affiliations:** 1 College of Veterinary Medicine, Huazhong Agricultural University, Wuhan 430070, China; farhan092@webmail.hzau.edu.cn (M.F.R.); 2020gss@webmail.hzau.edu.cn (S.G.); lizangxi97@webmail.hzau.edu.cn (K.L.); qcx2000@webmail.hzau.edu.cn (C.Q.); farahijaz0788@webmail.hzau.edu.cn (F.I.); yanlisanhuo@163.com (Y.L.); 2 College of Veterinary Medicine, Sichuan Agricultural University, Ya’an 625014, China; moquan@mail.hzau.edu.cn; 3 Hubei Jiangxia Laboratory, Wuhan 430200, China

**Keywords:** microplastics, nanoplastics, one health, livestock, poultry, animal-derived foods, exposure pathways, veterinary health, food safety, risk mitigation

## Abstract

Background: Microplastics (MPs) and nanoplastics (NPs) are now common in land and water ecosystems. Their spread is an increasing issue from a One Health perspective. These particles end up in soils, water, air, and farm inputs. This poses direct risks to animal health and indirect risks to people who eat animal-derived food. There are also risks from plastic additives and pesticides migrating with these particles in animal-based food. Scope and Approach: This review summarizes how MPs and NPs move in agroecosystems and livestock production. It covers their main sources, such as agricultural plastics, sludge-amended soils, plastic-lined storage, and environmental fallout. It explains how farm animals are exposed, including through feed, water, soil contact, and inhalation. Evidence is condensed for occurrence in manure, tissues, and animal products. The review also highlights key analysis challenges, especially those limiting the assessment of nanoplastic exposure. Key Findings: Field surveys show very different contamination levels in the environment. Agricultural soils range from 0.36 to 42,960 particles/kg. Livestock indicators, like contaminated feed and manure, range from 10^2^ to 10^5^ particles/kg. In free-roaming systems, chicken feces have very high loads, showing trophic transfer in land food chains. A pilot study found plastic particles in pig and cow blood, suggesting some particles cross the gut into the blood. Experimental models link MPs/NPs to oxidative stress, inflammation, mitochondrial dysfunction, metabolic disturbance, and potential reproductive toxicity in livestock and poultry. Conclusions and outlook: Animal-based foods provide a major source of human exposure. MPs and NPs have been observed in milk and poultry products, such as packaged meat and eggs (mean 11.67 ± 3.98 particles/egg). There is still a research gap on raw milk taken directly from the teat and on raw eggs that have not been handled or packaged. This gap makes it hard to identify real contamination sources and control strategies. The review stresses the need for harmonized detection methods (especially for NPs), monitoring from farm to fork, and practical ways to reduce plastic use on farms and minimize contamination during processing, feed handling, and packaging.

## Simple Summary

Microplastics (MPs) and nanoplastics (NPs) are widespread environmental pollutants, present in soil, water, air, and agricultural products, which are a significant risk to animal and human health. These microscopic-sized plastic particles can be ingested by farm animals via feed, water, and inhalation and accumulate in their products, such as milk, meat and eggs. The result of this exposure is oxidative stress, inflammation and metabolic imbalances in livestock and this may impact the health and productivity of animals. Moreover, these pollutants can be transferred through the food chain, potentially posing risks to human health. The paper mentions the importance of more effective detection methods and approaches to decrease plastic pollution in food systems and agriculture. A One Health approach, that is, linking human health with animal health and the health of the environment, will allow us to create more effective tools to manage and reduce the dangers posed by plastic contamination. This article highlights the importance of handling the issue of plastic pollution in agricultural fields to protect food safety and human well-being.

## 1. Introduction

The modern world is full of plastics and they are not coming out of the water or the earth. Rather than decomposing into biodegradable materials, the plastic materials break down into smaller and smaller pieces. These will eventually become microplastics (MPs; 0.1–5000 µm, i.e., 0.1 µm–5 mm) and nanoplastics (NPs; approximately 1–100 nm), collectively referred to as micro- and nanoplastics (MNPs) in this review. This relentless disintegration has caused the proliferation of micro- and nanoplastics (MNPs) all over the world. The physicochemical characteristics of MNPs comprise size and shape, polymer, and surface chemistry. Collectively, these properties determine their fate in the environment and modulate associated biological reactions.One of the toxicity mechanisms is the physical effect of the particles themselves. However, this mechanism is not as straightforward in the case of MNPs, due to their particulate nature. Plastics are a complex compound of complex polymers as well as a rich assembly of chemical additives. They include plasticizers, leftover monomers, flame retardants, antioxidants, UV stabilizers, colorants and pigments, most of which are tenacious to the environment and can be toxic. The others are termed as substances of very high concern (SVHCs) or legacy persistent organic pollutants (POPs). These have been the cause of significant environmental and human health concerns due to their disrupted endocrine and bioaccumulated effects on the long-term environment [1]. Besides the above-discussed toxicity mechanisms, there are other biological actions of micro- and nanoplastics (MNPs) caused by environmental corona formation on surfaces. Once in natural ecosystems, MNPs quickly obtain a dynamic co-layer of adsorbed organic and inorganic substances, like biomolecules (proteins, lipids, and polysaccharides), as well as a diversity of pollutants and microbial agents. This corona greatly changes the physicochemical identity of the particles. It affects their behavior in the environment, their bioavailability, and their interaction with biological systems. The corona is not homogeneous or fixed in composition. It changes over time due to environmental aging and local physicochemical factors, adding yet another complexity to MNP toxicity. Owing to this, MNPs represent a very heterogeneous group of contaminants. They have numerous context-dependent pathways of toxicity that challenge toxicological testing and environmental risk assessment [2,3,4]. Plastics are synthetic polymeric materials that have become part of contemporary life because of outstanding properties like resistance to corrosion, structural elasticity, low density, and affordability. These properties have accelerated and increased the use of plastic components in many industries. Plastics are used extensively in domestic utilities, agriculture, food wrapping and preservation, industrial processes, textile and apparel, large-scale manufacturing, and engineering uses [5]. Although plastic use is increasing worldwide, end-of-life management remains underdeveloped. Only about 9 percent of the plastic ever generated is estimated to have been recycled globally. Only 14 percent of plastic containers enter official recycling channels. The vast majority (between 79% and 91%) is discarded in landfills, burnt, or enters the environment. Recent statistics show that over 300 million tons of plastic waste is produced every year, much of which is not disposed of properly. Poorly handled plastic waste, especially where no proper waste collection or treatment exists, spills into land and water. It becomes stuck and propagates because it resists degradation. These materials move to freshwater and marine environments via wind, runoff, and wastewater, contributing greatly to global plastic pollution, especially in oceans and coastal systems [6,7,8]. Due to their adaptability and convenience, plastic products have become ingrained in contemporary society. They are now indispensable at both personal and industrial levels [9]. Nevertheless, the consumption of plastics combined with poor disposal habits and lack of effective waste disposal infrastructure has seen the massive build-up of plastic debris within the earth, in the atmosphere and in the ocean. The ecological effects of this contamination are also becoming clearer, with an ever-growing body of knowledge pointing out the negative effects of plastic waste on wildlife, both through physical entrapment, ingestion, and through exposure to toxic materials related to plastic waste [10]. Gradually, macroplastic waste is converted to microplastics and nanoplastics by physical mixing and abrasion, chemical degradation, and biological life. These smaller particles form as larger plastic components disintegrate. They are considered secondary microplastics and nanoplastics. In contrast, primary microplastics and nanoplastics are intentionally produced in small sizes for commercial, industrial, and consumer products. Examples are microbeads in facial cleansers and toothpaste, abrasives in industrial cleaning products and pre-production resin pellets, also known as nurdles, in the manufacture of plastics [11,12]. Micro- and nanoplastics (MNPs) occupy all large areas of the environment such as surface waters, sediments, ground water, soils and the atmosphere. MNPs have extensively appeared in drinking water as well as other trophic levels of the food web and even more so in the agri-food chain, and this has created great concern regarding the ecological integrity and health of people [13,14]. It is estimated that 1.15 to 2.41 million metric tons of plastic waste is flowing into the oceans every year. This is approximately 80 percent of land waste. Such high levels of disposal of plastic waste point out the necessity of enhancing waste management on land. They also demonstrate the vitality of the efforts of actions on the ground to solve marine plastic pollution [15]. Microplastics are generally defined as solid polymer particles smaller than 5 mm. For consistency, and in line with the EFSA working definition, we define microplastics (MPs) as particles with at least one dimension between 0.1 and 5000 µm (0.1 µm–5 mm) and nanoplastics (NPs) as particles approximately 1–100 nm (0.001–0.1 µm) [16]. Particle size is a critical determinant of environmental transport, bioavailability and toxicokinetics; therefore, these size boundaries are applied consistently throughout the manuscript. MPs/NPs have been detected in multiple environmental matrices (freshwater, drinking water, air and food) and can be taken up by organisms across food webs, raising concerns for animal and human health [17]. The size is the main difference between microplastics and nanoplastics, and to a certain degree, a source. The typical plastic objects are referred to as microplastics, including fragments, fibers and beads, which have a diameter less than 5 millimeters (mm) with certain classifications defining them as less than 1 micrometer (µm) in diameter. Nanoplastics, conversely, are generally described as substances less than 1000 nanometers (nm) in diameter, but even narrower definitions exist of 1–100 nm. The exact limits between these two groups are still considered issues of scientific debate, primarily because of the current problems related to the detection, quantification and characterization of plastic particles at the nanoscale [18,19]. Unfortunately, only about 9 percent of all plastic waste is recycled worldwide. The remaining 91 percent ends up in landfills, is burned, or is dumped into the environment, causing widespread and long-term pollution across ecosystems [20]. Microplastics are presently being accepted as ubiquitous pollutants in nearly every environmental matrix. These matrices include freshwater systems, drinking water sources, atmospheric air, food products, and both aquatic and terrestrial ecosystems. Worryingly, microplastics have also been confirmed to be present in the human body, including the placenta and fecal matter. These plastic materials have a wide size scale, spanning from approximately 100 nanometers (nm) to 5 millimeters (mm). Nanoplastics are even smaller, going under 100 nm in at least one direction. The large presence of micro- and nanoplastics is concerning because they are highly likely to be taken up by a wide range of organisms. This includes organisms at the bottom of food webs. Furthermore, they present contemporary ecological and a few possible human health hazards [21]. Not only have microplastics have emerged as a pervasive pollutant in almost all environmental matrices, freshwater, drinking water, air, food, and aquatic and terrestrial environments, but recent research has also validated that there are microplastics in the human body, including in the placenta and fecal matter. The size of these plastic particles is between one hundred and fifty nanometers (nm) down to 5 millimeters (mm), whereas nanoplastics are those that measure less than 100 nm in at least one regard. The universal spread of micro- and nanoplastics is especially problematic since they have a high potential of being absorbed by various organisms including the low-level organisms of food webs. In addition, these particles are dangerous to ecology and can have serious health-related risks to human beings [22]. The persistent accumulation of micro- and nanoplastics (MNPs) in the environment is expected to result in increasing concentrations, thereby elevating the risk of exposure across biological systems, including plants, animals, and humans. To date, most research on MNPs has focused on aquatic and terrestrial systems, particularly examining various invertebrate and vertebrate species, with recent attention directed toward human exposure and associated health effects. A comprehensive understanding of the risks posed by MNPs and the development of effective preventive strategies require the adoption of a One Health approach. This integrative framework recognizes the interdependence of human, animal, and environmental health and promotes multidisciplinary collaboration to advance these domains in a sustainable and balanced manner [23,24]. Animals, both cattle and fowls, are part of the agricultural systems of the whole world. These animals are essential to the realization of food security and the livelihood of rural communities as well as the development of the economy. Livestock can be explained as domestic mammals like cattle, sheep, goats, pigs, and horses, while poultry are domestic birds like chickens, ducks, turkeys and pheasants [25,26]. Plastics have been a common household feature in modern society since the invention of Bakelite at the beginning of the twentieth century, which is derived through the use of petrochemicals. The use of plastics is now very extensive in industrial and domestic fields [27]. Passage of manufacturing plastic on an international level has increased manifold. Consumption increased to more than 367 million metric tons in 2020, compared to just over 1.7 million metric tons in the 1950s. In the meantime, improperly handled plastic waste has increased, with some estimates showing it to be over 218 million tons of garbage per year and it poses a significant worldwide environmental and waste problem [28,29]. Plastic waste poses a threat to the environment and gradually turns into microplastics (MPs, less than 5 mm) and nanoplastics (NPs, less than 100 nm). Such transformation occurs by environmental forces that include ultraviolet (UV) radiation, mechanical weathering and the action of microbial activity. Those particles have significantly saturated land and water organisms. They have polluted the majority of the world through the soil, water and air [30,31,32]. Microplastics (MPs) and nanoplastics (NPs) are present both in the products of livestock and poultry, including dairy meat, milk, eggs and animal manure. The results of the research cause alarm regarding the impact of plastic particles on human beings via the food chain. There is a need to conduct more research about their effects on food safety and human health [33,34,35]. Farm animals can be exposed to microplastics and nanoplastics (MNPs) in three ways: oral intake, inhalation, and dermal intake. These particles may accumulate in animal tissues, and they can present a number of physiological effects. Although the routes of exposure have been well-established, it is important to pay attention to the environmental variability and dose–response relations when assessing their risks [20,36,37]. Humans and animals are both becoming more exposed to micro- and nanoplastics (MNPs). Growing evidence supports the notion that core toxicological mechanisms, including immunomodulation, oxidative stress, and genotoxic damage, show substantial conservation across species. Nevertheless, farm animals have remained largely underrepresented in MNP studies through the One Health construct. This underrepresentation persists despite their central role in the agriculture and food system. Livestock can act as vectors and paths of MNPs. These contaminants can also contribute to human exposure via consumption of animal products, including milk, meat, and eggs. Given the escalating environmental burden of plastic pollution, farm animals may experience near-constant exposure. This raises concerns about possible effects on their health, such as reproductive ability, immune capacity, and productivity. Therefore, farm animals could be important sentinels to study the health hazards of MNP exposure in humans. This also highlights the need to integrate cross-species toxicology research within the framework of One Health [38]. In this review, we briefly survey new evidence concerning micro- and nanoplastics (MNPs) in agriculture. We cover their sources, stages and levels of exposure, and possible effects on farm animal health. We emphasize the urgent need to better define the presence, distribution, and migration of MNPs through farm systems. This includes environmental inputs, animal feed, livestock, and the transfer to people via animal products. Adopting a One Health approach, we propose that health is interactively linked among animals, humans, and ecosystems. Finally, we identify the main knowledge gaps and offer specific recommendations for future studies to better understand and reduce MNP contamination in agriculture.

## 2. Review Methodology

The variety of study designs (field surveys, laboratory experiments, and mechanistic in vitro studies) and the diversity of methods of analysis allow for the presentation of this work as a narrative review supported by a systematic literature search (but not a formal systematic review or meta-analysis). Databases and search strategy: PubMed/MEDLINE, Web of Science Core Collection, and Scopus were searched and Google Scholar was also screened to find relevant gray literature and recent publications that are not yet included in bibliographic databases. Database searches were carried out until 30 June 2024 and reference lists of significant reviews and highly relevant primary studies were searched by hand to identify more papers. Search terms: We used controlled vocabulary (where possible) and free-text terms associated with plastics and agricultural/veterinary uses. Example query blocks are: (microplastic OR micro-plastic OR nanoplastic OR nano-plastic OR one health) AND (soil OR water OR air OR farm or agriculture OR exposure OR uptake OR toxicity OR inflammation OR oxidative stress). Inclusion and exclusion criteria: We identified articles with the following criteria: (i) comprising micro- and/or nanoplastics in the agricultural environment, livestock/poultry, manure, or animal-derived foods or (ii) measuring toxicological mechanisms or health-relevant outcomes in vertebrate models with clear characterization of the particles. Only studies that included marine organisms and were not related to farm systems or animal-derived food were excluded together with those that did not contain adequate information on identifying/characterizing the particles. Selection and synthesis of studies: The selection and synthesis of the studies was done by screening the titles/abstracts and assessing the remaining qualifying articles based on the full text. The synthesis of evidence was done qualitatively and categorization was based on exposure route, biological compartment, and health endpoint. Where we found two studies answering the same question, we prioritized the one with a more defined particle characterization (e.g., FTIR/Raman and pyrolysis-GC/MS where needed), clearer QA/QC controls to minimize contamination, and increased relevance to livestock production systems. Significant precedent studies and key guidance documents were kept if they influenced definitions, analytical restrictions or conceptualization.

### 2.1. Pathways of Exposure and Uptake of Micro- and Nanoplastics (MNPs) in Livestock at the Farm Level

**Figure 1:**
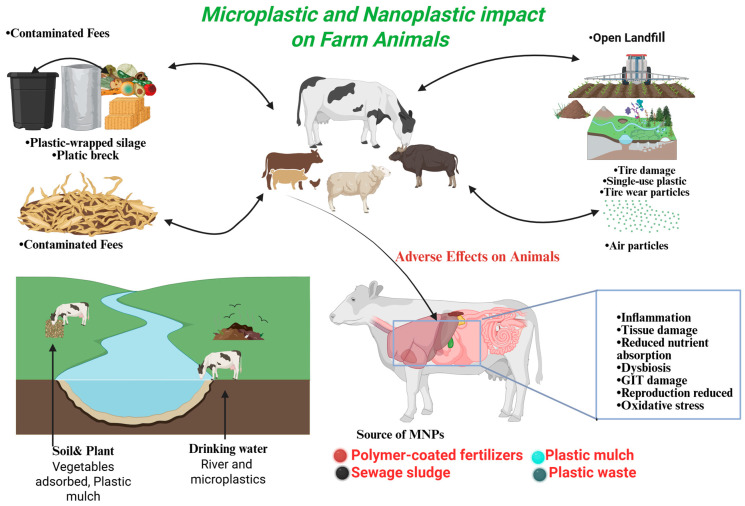
Pathways and adverse effects of microplastics and nanoplastics (MNPs) on farm animals.

The growing rate of microplastic and nanoplastic (MP-NP) pollution is a major threat to the entire system of livestock and poultry production worldwide. Micro- and nanoplastics have appeared in all kinds of animals and environments. Nevertheless, it is not clear how many of these exposures can be converted into deleterious systemic consequences in the real world, and this is what has been studied. Empirical investigations have proven that there is MP-NP particle content in animal feed and drinking water as well as air particulates, which is a clear indication that farmed animals can be exposed both via ingestion and inhalation. This ongoing environmental footprint is extremely dangerous to the well-being of the animals, food safety, and proper handling of livestock production in a sustainable manner [39,40]. Microplastics (MPs) have been found to build up in the animal gastrointestinal tract when ingested and may cause a continuum of adverse health effects, including inflammatory conditions, reduced nutrient uptake, and internal tissue damage. Nanoplastics (NPs), which are particles less than 100 nm in diameter, are now being identified as an environmental contaminant of concern. Their size, as well as bioavailability, affect their possible health effects significantly [41,42]. Besides livestock, there are reports of microplastics and nanoplastics being found in marine invertebrates, fish and aquatic mammals. To illustrate, marine invertebrates such as mussels and fish species like trout and bass have demonstrated that they accumulate plastic particles from polluted waters in large amounts, with effects on their health and the ecosystems they maintain [38]. Plastic materials have broad uses in agricultural systems in the form of various applications that increase the productivity and the efficiency of the operations. The uses of these applications are polymer-coated fertilizers, plastic foil wraps, fruit protection foams, and silage wraps to preserve grass and airtight forage. Also, animal feed is likely to be stored in silos or in unsealed plastic bags, thus enhancing the risk of microplastic and nanoplastic (MNP) contamination. The second notable cause of MNPs in agricultural soils is the application of sewage sludge as a fertilizer, which further facilitates the extensive spread of plastic particles on agricultural lands [43,44,45]. Micro- and nanoplastics (MNPs) have become a problem in soil. Their emergence is recognized as a contaminant of concern. The density of MNPs in agricultural soils varies greatly depending on regional practices, land use, and waste management conditions. Reported concentrations range from 0.36 particles per kilogram in southern German agricultural fields to 42,960 particles per kilogram in some places in China. The findings led to the United Nations Environment Programme (UNEP) being concerned over the danger of plastics deteriorating in farmlands. The UNEP pointed out how this contamination would affect food security, particularly with the threat this contamination has on the health and fertility of the soil [46]. Micro- and nanoplastics (MNPs) can be found in several environmental exposure routes for farm animals as they graze due to ingestion of contaminated surface water and oral uptake of the micro- as well as nanoplastics bound or adsorbed to plants. Other recent studies have also shown that MNPs accumulate in plant species, resulting in the possibility of herbivorous livestock being exposed to MNPs through such a route [47]. A study that was carried out in southeastern Mexico monitored free-range chickens foraging through household gardens that were polluted with plastic pollution along traditional Mayan settlements. The study presented an average content of plastic particles of 870 particles/kg of soil, 14,800 particles/kg of earthworm casts and 129,800 particles/kg of chicken feces. These results are highly indicative of bioaccumulation and possible trophic transfer of microplastics in land-based food webs [48]. The concentrations of microplastic in agricultural fields in the areas where sheep graze have been calculated in the southern parts of Spain and have been reported to reach a minimum of 2000 particles per kilogram of soil. This discovery shows there are possibilities of plastic build-up amongst pastoral farming systems [49]. A survey carried out in southern China found that there was rampant contamination of livestock feed with plastic debris, which included chicken, pig, and cow feed. The researchers determined the number of wet chicken feed particles in chicken food to be 96 to 109 per kilograms, pig food to be 115 to 139 per kilograms and cow food to be 360 to 630 per kilograms. The pollutants included all types of polymers and polyethylene (PE) was the dominant one. PE was present in pig feed, cow feed (100 percent), and chicken feed (about 30 percent) as a proportion of plastics. These results point to a high possibility of being exposed to microplastics by way of livestock nutrition and demonstrate the urgent need to enhance the quality of the feed consumed by farm animals [50]. The concentrations of the micro- and nanoplastics (MNPs) in the agricultural system and in animals that have been reported are highly variable over the published studies. To some extent, this disparity can be explained by the lack of standardized cross-validated analysis techniques. Moreover, it is difficult to detect and measure nanoplastic (NP)-containing particles because they are very tiny. However, existing studies could offer meaningful initial evidence on MNP contamination in food production and livestock. In order to address constraints with NP detection, researchers are actively adopting computational approaches to predict the abundance of NPs, especially with enhanced quantification of larger microplastics (MPs). To give an example, a principal component analysis (PCA)-based algorithm that combines both Raman spectroscopy and scanning electron microscopy to determine the amount of Teflon-derived MNP particles has been presented by Luo et al. This methodology has the potential to be used to measure NP concentrations when direct measurements are not possible [51]. Micro- and nanoplastics (MNPs) have been identified in the manure of farm animals, such as sheep, pigs, chickens, and cows, at concentrations ranging from 10^2^ to 10^5^ particles per kilogram. This evidence demonstrates that livestock are exposed to and ingest MNPs in real-world agricultural environments. Furthermore, MNPs have been detected in the digestive tracts of sheep, confirming that these particles are ingested and retained within the gastrointestinal systems of grazing animals [52,53]. A recent pilot study identified plastic particles in the blood of pigs and cows, thereby enhancing current knowledge of micro- and nanoplastic (MNP) bioavailability in livestock. MNPs in blood sources are indicative of the fact that some particles can penetrate the intestinal barrier and enter the bloodstream after oral administration rather than being excreted as feces. Although most of the ingested MNPs might still stay in the digestive system, such a finding suggests internal distribution and the need to understand more about the accumulation of particles in tissues and potential systemic impact on the health of animals [54]. Mouse investigations have shown that orally delivered pristine polystyrene (PS) micro- and nanoplastics (MNPs) build up within numerous bodily systems, which encompass the lungs, spleen, liver, kidneys, intestines, brain, and reproductive organs. These results suggest that after gastrointestinal absorption, MNPs may be disseminated throughout the body and this poses questions of their long-term toxicological impacts [55,56]. Given the similarity of uptake and transport pathways among vertebrates, rodent studies indicate that micro- and nanoplastics (MNPs) are capable of crossing biological barriers and accumulating in the tissues of other organisms, including livestock. Nevertheless, targeted monitoring and toxicological investigations in farm animals are required to elucidate the consequences of chronic MNP exposure, particularly in light of their distinct physiological characteristics. For instance, ruminants such as cows possess a complex ruminal microbial ecosystem that may degrade or modify certain MNPs, thereby influencing their bioavailability, toxicity, and distribution [57]. The presence of polyethylene (PE) in cow feed and polypropylene (PP) in their manure implies that there is a potential transformation, selective retention, or degradation of polymer in the gastrointestinal tract. These results highlight the necessity of learning the behavior of micro- and nanoplastics (MNPs) in vivo. MNPs are dangerous in chemical, physical and microbiological terms and more than 10,000 leachable plastic compounds have been identified, of which about 2400 are toxicologically hazardous. The above insights underscore the necessity of completing a holistic risk evaluation of plastics along the food chain to protect the health of animals and people [58]. As shown in Figure 1.

This diagram gives an overview of the environmental pathways via which microplastics and nanoplastics find their way into the food chain of farm animals and how they ultimately affect them physiologically. Large sources of exposure are contaminated feed, silage wrapped in plastic, plastics, contaminated drinking water (rivers and runoffs), plastic mulch on the soil, and open landfills which release airborne plastic particles. After being ingested or inhaled, these particles build up in effect in animal tissues and lead to numerous health consequences such as gastrointestinal inflammation, tissue damage, malabsorption of nutrients, gut microbiota dysbiosis, oxidative stress, and impaired reproductive function.

### 2.2. Human Exposure to Micro- and Nanoplastics Through Animal-Derived Food Products

**Figure 2:**
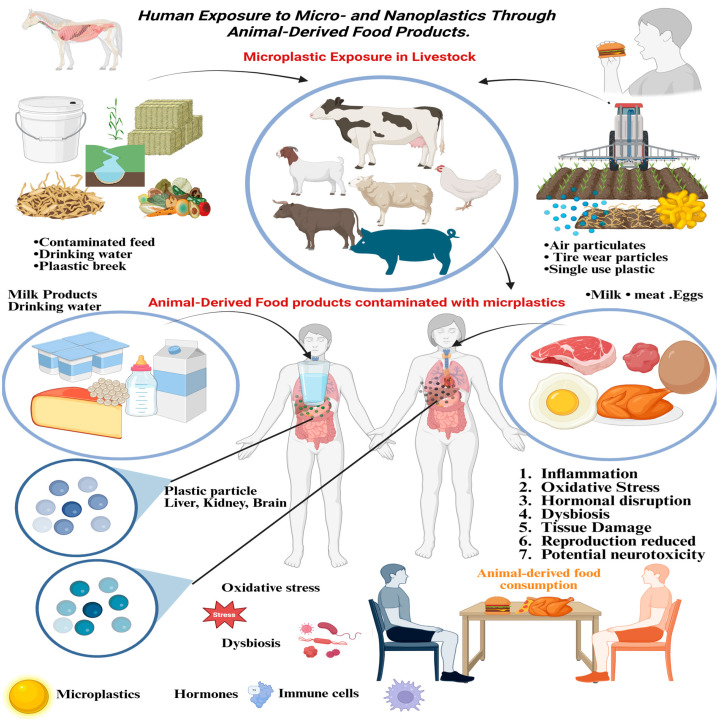
Human exposure to micro- and nanoplastics via animal-derived food products.

In addition to the possible health effects that micro- and nanoplastics (MNPs) have on farm animals, they are a direct pathway of exposure in the human body since they are found in animal-based food stuffs. Recent findings have discovered the presence of MNPs in various dairy products such as processed, liquid, and powdered milk products. These findings bring to the fore rising concerns of how plastic residues are being introduced into the human food chain through the production systems of livestock [59,60]. Several sources are attributed to the existence of micro- and nanoplastics (MNPs) in milk. MNPs can get into cows via contaminated air, feces, and forage. Further contamination may happen in the milking and dairy processing chain, in pre-farm milking, mechanical processing, transfer, and further milking processing, which includes filtration, pasteurization, and storage. The packaging process is also another possible source of contamination in the milk processing plant. At every step of this chain, there are possibilities of plastic particles getting into the final dairy product [61]. Recently, a research survey found that polyethylene (PE) was the most common plastic polymer in milk and was in direct relation to the storage tanks that were on the farm. The results show that PE contamination was probably a result of milking equipment. The introduction of microplastics into raw milk can be helped by mechanical abrasion or shedding of the material over the course of the milking process [62]. Polyethersulfone (PES) is one of the most common polymeric plastics that are found in milk samples. It is often used in membrane filtration systems, which are widely used in dairy and food processing industries. PES presence in milk is probably explained by the shedding or elution of particles over the course of filtration [63]. One of the important areas of the human diet is dairy products and their plant-based equivalents. The identification of micro- and nanoplastics (MNPs) in dairy products has elicited a lot of concerns about the effects that the products may have on human health. Indicatively, in the Netherlands, dairy products (333 g per capita, 2012–2016) were the most eaten foodstuffs, as compared to other products. Such a high exposure highlights the necessity of researching the possible MNP exposure via dairy production and conducting additional studies on the pathways of contamination and the related health responses [64]. Similarly to dairy products, meat may also serve as a source of micro- and nanoplastic (MNP) exposure. A study conducted in Mexico identified MNPs in the gizzards of poultry feed intended for human consumption. These results indicate that edible animal tissues have the potential to be contaminated with plastic particles [48]. Recent studies have detected micro- and nanoplastics (MNPs) in packaged poultry meat, suggesting that contamination can occur during processing, packaging, or storage within the food production chain [65]. Plastics are also delivered to land animals such as the wild boar and deer via contaminated feed, water and soil. Microplastic contamination has been found in the wild environment and this indicates that the presence of these pollutants is not limited to farmed animals but extends to the environment in general [66]. Micro- and nanoplastics (MNPs) may enter animal-derived foods through two primary pathways: biological uptake and systemic distribution within the animal, or contamination during food processing. To date, no research has directly examined the presence of MNPs in raw milk collected directly from the udder or in freshly laid eggs prior to any processing, handling, or packaging. This lack of investigation results in a significant knowledge gap regarding the principal sources of MNP contamination within the food production chain. Targeted research is needed to identify and define critical control points for MNP introduction, including specific stages from production within the animal to the final product reaching consumers, as described in Figure 2.

This figure shows the flow of microplastics (MPs) and nanoplastics (NPs) in the environment to human beings via livestock. Animals kept on farms are exposed to MPs through contaminated feed, water and particles in the air. The particles are deposited into the animal tissues and passed on to humans when they consume the contaminated products such as milk, meat and eggs. The accumulated MPs can also be localized in critical organs (liver, kidney, and brain), which can lead to inflammation, oxidative stress, hormonally disturbance, dysbiosis, tissue damage, reproductive dysfunction, and neurotoxicity.

#### Considerations of Study Quality and Bias

In this review, we identify that the experimental design, particle nature and dose relevance of the included studies are heterogeneous. Numerous in vitro and in vivo tests employ uncontaminated polystyrene balls that do not necessarily indicate the complexity of agricultural system microplastics (MPs) and nanoplastics (NPs). As well, exposure levels tend to be greater than those found in the field. Although we did not use a formal risk-of-bias tool, we qualitatively classified the studies mentioned according to the following parameters: (i) particle type (pristine vs. environmental), (ii) exposure dose relevance, (iii) biological model (rodent, poultry, fish, and cell line), and (iv) outcome validity. Artificial exposure studies that were more in line with realistic exposures and type of particles were identified as more representative in terms of risk assessment. We also acknowledge the potential for publication bias in the toxicological literature, where studies showing negative or null effects may be underrepresented. Readers are advised to interpret reported health risks with attention to the methodological strengths and limitations discussed herein. Evidence strength is also different based on the study design. In vitro experiments have mechanistic information but are not complex systems. Animal experiments in vivo give informative results on the physiological response, but care must be taken when applying the results to humans because of interspecies differences. Observational studies in the agricultural environment offer practical applicability, but they may not be able to control confounding factors. These differences enable readers to understand the strengths and useability of the mentioned results in the proper way by pointing out the differences. The effects of these microplastics are summarized in Table 1.

### 2.3. Detrimental Impacts on Gastrointestinal Function and Metabolic Regulation

**Figure 3:**
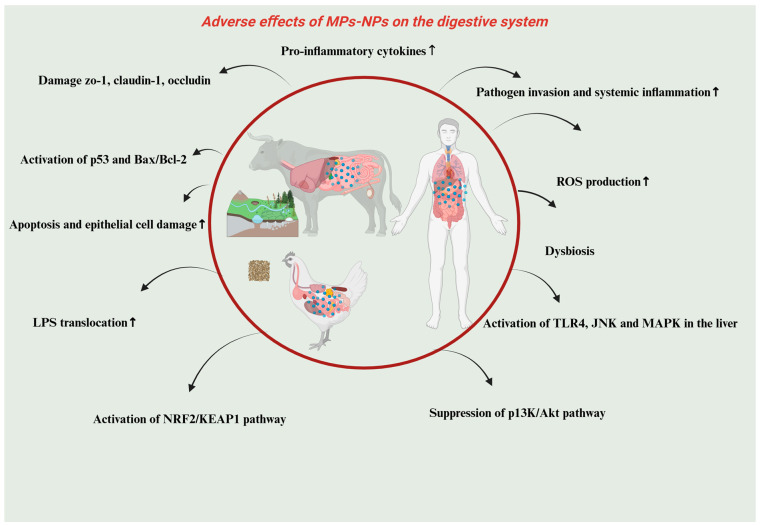
Adverse effects of microplastics and nanoplastics (MPs–NPs) on the digestive system.

Microplastics (MPs) and nanoplastics (NPs) are recent contaminants that are impacting the earth, and more and more studies are proving their adverse effects on the gastrointestinal system and metabolism of mammals and other species. The consumption of MPs and NPs can cause considerable gastrointestinal dysfunction, impaired nutrient absorption, and changes in microbiota composition of the gut, as well as derailing metabolic homeostasis in the farm animals. Although these studies provided great mechanistic information, it must be noted that most of them were done at concentrations that are more than those that are environmentally relevant. Therefore, one should be careful about generalizing these findings to farm animals or humans in a normal field environment. The interpretation of the observed toxicological results should be in relation to dose, route of exposure and species differentiation. In fact, the vast majority of laboratory experiments are conducted with high-dose, acute exposures with pristine particles which do not represent chronic, low-dose, and complex exposures to environmental MNPs. Therefore, these models produce important mechanistic information, but a great deal of care must be exercised when generalizing these findings to farm conditions or community health conditions, unless exposure modeling is done. Even though most of the current research has been done in rodent and avian models, the toxicological findings that have been observed are valuable in understanding the risks that these ongoing plastic pollutants may cause to livestock in the future. Due to the high environmental distribution of MPs and NPs, there is an urgent need to conduct more research on farm animals and analyze their particular susceptibility and implement adequate measures to mitigate it [82,83]. A recent study used four-week old mice that were exposed to polystyrene nanoplastics (0, 0.2, 1, and 10 mg/kg body weight) orally over 30 days. The exposure changed the expression of genes related to mucus and changed the composition of the gut microbiota but did not produce changes in behavior. Also, inflammation, oxidative stress, and tissue damage were not significantly observed in the major organs or in the blood serum, which implied a limited toxicological impact in the represented conditions [84]. It has been revealed that microplastics (MPs) undermine the structural and functional integrity of the intestinal barrier, which makes it more vulnerable to the development of inflammation, increases intestinal permeable characteristics, and decreases the level of mucus secretion. Through experimental research, it has been proven that exposure to MPs may result in reduced mucus secretion and destruction of the intestinal epithelial layer. All of these changes are harmful to the functioning of the gut barrier, which may be conducive to the entry of maladaptive pathogens and toxicants into the systemic circulation, with consequences to host immunity and overall well-being [82]. In addition, microplastics (MPs) were also assessed to disrupt the expression of the genes linked to the modulation of intestinal tight junction proteins, thus aggravating the integrity of the epithelial barrier. This disturbance augments the heightened intestinal permeability and the onset of pro-inflammatory responses, planting the seeds of gut dysfunction [85]. Intestinal tissue necrosis and pyroptosis in avian models have been linked to polystyrene microplastics (MPs). Such pathological alterations are accompanied by the breaking of the gastrointestinal vascular barrier, which helps to move toxins and other harmful elements to the bloodstream, further increasing the general physiological dangers [86]. Such negative outcomes are specifically pronounced in poultry species, where contact with microplastics (MPs) can undermine the integrity of the intestines, making them susceptible to poor health and productivity, poor nutrient absorption and ultimately systemic infections. A growing body of evidence has shown that MPs have a far-reaching effect on the gut microbiota, which causes dysbiosis and metabolic imbalances. In particular, exposure to MPs has been associated with decreased viable populations of beneficial microbes, including Firmicutes and Actinobacteria, and increased the expansion of potentially pathogenic microbes, including Proteobacteria. These microbial alterations may influence immune regulation, nutrient metabolism, and disease susceptibility in exposed animals [87,88]. Microbial dysbiosis may cause inflammation and impact digestion and energy metabolism. The modified gut microbiota influences bile acid metabolism, lipid metabolism, SCFs, and amino acid metabolism, which are significant processes in the maintenance of metabolic equilibrium. These disturbances can lead to more widespread physiological disequilibrium, affecting the immune system, development and the health of the animals [82,89]. Microplastic (MP) exposure in farm animals has an especially alarming metabolic implication since exposure can cause malnutrition, impaired immune performance, and reduced productivity, which have an ultimate impact on agriculture efficiency and food safety. In addition to the gastrointestinal tract, MPs and nanoplastics (NPs) have been reported to disrupt systemic metabolism, which results in oxidative stress and mitochondrial dysfunction in critical metabolic organs. Research has established that MPs are capable of accumulating in the liver, leading to oxidative damage, lipid build-up and interference with normal hepatic metabolic processes, which may lead to impaired energy balance as well as detoxification pathways [76]. In terrestrial animals, the liver is the main center of nutrient metabolism, xenobiotic detoxification, and systemic energy homeostasis regulation. This means that any interference with hepatic functionality (induced by microplastics (MPs)) would have long-term consequences with regard to animal health, development, and production. There is also emerging evidence that MPs are able to interfere with the glucose and lipid metabolic pathways, and this may lead to the development of metabolic diseases. The same metabolic imbalances have been recorded in rodent models fed with MPs in high-fat diets, and again, this supports the likelihood of MPs causing metabolic imbalances in livestock and other farmed species [90]. Exposure to microplastics (MPs) has been associated with metabolic imbalances including insulin resistance, liver fat build-up and enhanced pro-inflammatory cytokines, which predispose individuals to conditions like fatty liver disease. MPs can also interfere with the gut–liver axis and permeate through the intestinal barrier, initiating immune responses and low-grade inflammation which lead to more widespread metabolic and hepatic malfunction [86]. Exposure to microplastics (MPs) has been linked to high concentrations of lipopolysaccharides (LPSs), which are one of the major constituents of the outer membrane of Gram-negative bacteria. Once introduced into the systemic circulation, LPSs can elicit chronic low-grade inflammation and hence play a role in metabolic derangements and immune dysregulation [89]. Exposure to microplastics (MPs) has been associated with elevated levels of lipopolysaccharide (LPS), a major component of the outer membrane of Gram-negative bacteria. Once LPS enters the systemic circulation, it can promote chronic low-grade inflammation, thereby contributing to metabolic disturbances and immune dysregulation [90]. Research has revealed that microplastics and nanoplastics induce oxidative stress, inflammation and mitochondrial dysfunction in different organ systems. Nevertheless, the precise health hazards are still unclear because the characteristics of the particles and exposure doses are different. Future research should be conducted to standardize exposure requirements and investigate the long-term effects at environmentally relevant concentrations [77,91]. The heterogeneity of the toxicological literature is also significant with respect to study design variability (differences in particle size (less than 50 nm to several µm), polymer type (e.g., PE, PS, and PVC), route of exposure (oral, inhalation, and dermal), and dose. The abundance of many of these is far beyond environmentally relevant levels, making it harder to extrapolate the findings and make them ecologically pertinent. This heterogeneity is to be taken into account when generalizing results or making generalized conclusions. Mitochondria play a central role in cellular energy metabolism and impairments in livestock and poultry may hamper the growth and feed efficiency and heighten vulnerability to metabolic disorders. Microplastics (MPs) are also known to enhance adipogenesis, which could change body composition by promoting fat deposition. In a study involving the use of fluorescent polystyrene nanoplastics (80 nm; 5 and 15 mg/kg), the nanoparticles were found in organs such as the liver, kidney, spleen, and pancreas, showing organ-selective toxicity. Disturbed lipid metabolism and hepatic dysfunction were observed. Transcriptomic data showed a reduction in the genes of ROS generation and the PI3K/Akt pathway. Persistent exposure elevated the levels of ROS and blood glucose and increased insulin resistance by increasing IRS-1 phosphorylation and decreasing Akt activity [92]. As illustrated in Figure 3.

The following diagram describes the most important molecular and cellular damage caused by ingested MPs and NPs in livestock and humans. Exposure causes intestinal barrier dysfunction, oxidative stress (↑ROS), inflammation, and epithelial apoptosis and dysbiosis. Activation of TLR4, JNK, MAPK, p53, and NRF2/KEAP1 and inhibition of the PI3K/Akt signaling pathway are the involved mechanistic pathways. These disturbances are driving factors of systemic inflammation and pathogen susceptibility.

### 2.4. Pathophysiological Effects on Reproductive Organs and Hormonal Balance

The extensive dispersion of microplastics (MPs) and nanoplastics (NPs) into the environment has created rising dread about the impact of these materials on the reproductive physiology of animals, which has proven to be harmful. A lot of the experiments reported in this section utilized in vivo or in vitro models that were exposed to large doses of pristine polystyrene particles. Even though these models are plausible in their mechanisms of reproductive toxicity, such as oxidative stress and endocrine disruption, these results are not yet applicable to agricultural exposure in the real world. The longitudinal data of livestock and human epidemiology studies are currently inadequate and further studies are required to provide dose–response associations in realistic exposure conditions. It has been demonstrated that exposure to micro- and nanoplastic (MP/NP) may lead to dose-dependent reproductive toxicity via oxidative stress, endocrine stress, inflammation and genetic damage pathways. Livestock and poultry subjected to environmental MPs are the subject of concern regarding these effects. In rodent models, polystyrene microplastics (PS-MPs) have been found to accumulate in reproductive organs where they cause ovarian inflammation, reduced oocyte quality, and altered reproductive biomarkers. It is noteworthy that IL-6 is increased and glutathione (GSH) and mitochondrial activity are disturbed [55]. It has been demonstrated that disturbances in mitochondrial membrane potential and endoplasmic reticulum calcium homeostasis are likely to impair oocyte viability, which can result in decreased reproductive performance in livestock species. Also, microplastics (MPs) disrupt folliculogenesis in the ovary by disrupting hormonal pathways essential to the normal maturation and development of the follicle. These disruptions can compromise follicular physiology and oocyte physiology, which eventually influences the outcome of fertility and reproduction in impacted animals [93]. Micro- and nanoplastics (MNPs) can disrupt multiple sensitive reproductive functional areas and stages of early development, potentially undermining the health, viability, and resilience of future generations [94]. The existing evidence on the reproductive impact of micro- and nanoplastics (MNPs) has mainly been obtained through the use of aquatic organisms and soil invertebrates, and, more recently, a small range of studies have taken place in rodent models. Micro- and nanoplastics (MNPs) have been determined to cross blood–testis and blood–follicle barriers, and thus direct access is obtained to the microenvironment that encloses spermatozoa and oocytes. This has been established in rodent models, showing their potential to interfere with gametogenesis and reproductive [95]. In addition to the accumulation of polystyrene micro- and nanoplastics (PS-MNPs) in reproductive organs like the ovary and the uterus, there are emerging studies on the adverse effects on fertility following maternal exposure. It is remarkable that transgenerational effects are seen with maternal exposure to PS-MNPs during gestation and lactation, with a carry-over effect on the children. Such effects involve physiological behaviors that are different in the offspring as well as a high propensity to metabolic complications, which underscores the possible long-term risks of exposure to MNP to reproductive functions and development [96,97]. It has also been demonstrated that plastic particles pass through the blood–placenta barrier, as micro- and nanoplastics (MNPs) have been detected on the fetal side of the human placenta. Such a result is a cause of concern regarding possible fetus exposure and developmental risks [98]. Micro- and nanoplastics (MNPs) can penetrate through reproductive barriers, such as gametes, embryos, and the placenta, and can lead to exposure during the entire lifespan, starting at the point of conception. Nevertheless, their impacts on early development are not well known. Though rodents are very widely used as models in reproductive toxicology, farm animals, especially cattle, can be used as useful alternatives. A bovine embryo model, in which slaughterhouse-derived oocytes and IVF embryos have been used, has been effective in investigating periconceptional stress and may be a viable model of MNP-related reproductive and developmental toxicity [99,100]. The bovine embryo model is an interesting alternative to use in early reproductive development studies, as bovine and human reproduction have very significant physiological and developmental similarities. Cattle are mono-ovulatory, in contrast to rodents, and important aspects of the reproductive cycle, including estrous cycle length, follicular dynamics, time to oocyte maturation, and oocyte size, as well as the time of embryonic genome activation, are highly homologous to those of humans. These common features contribute to the improved translational importance of bovine models in the study of factors that determine oocyte competence and early embryogenesis [101]. In a recent study, the influence of polystyrene nanoplastics (PS-NPs) on the reproductive functioning of female Wistar rats was tested. Rats were injected with PS-NPs (0 1.5 mg/day, 0.5 um) for more than 90 days, and ovarian and serum samples were examined. PS-NPs at 0 25 mg/L, which included and excluded antioxidant N-acetyl-L-cysteine (NAC), lead to PS-NP internalization, fewer developing follicles, and less anti-Mullerian hormone (AMH) in vitro in granulosa cells. Exposure caused oxidative stress, granulosa cell apoptosis, and nodular fibrosis, associated with the stimulation of the Wnt/-catenin pathway and fibrotic markers, including TGF-0, fibronectin, and 0-SMA. Importantly, NAC co-treatment minimized oxidative stress, prevented apoptosis, and inhibited Wnt/catenin stimulation, indicating antioxidant effects in alleviating PS-NP-induced ovarian toxicity [102]. Research was conducted that aimed at assessing the effects of polystyrene nanoplastics (PS-NPs) on male reproductive health and the condition of the blood–testes barrier (BTB) in Wistar rats. PS-NPs (500 nm), which were administered to males in a control group of 32 rats, were administered to the animals at a daily dose of 0, 0.15, 0.0015, and 1.5 mg for 90 days. The results showed that exposure to PS-NPs resulted in severe structural damage to the seminiferous tubules, a significant rise in spermatogenic cell apoptosis, and a significant decrease in the motility of sperm and their concentration, as well as an increase in the percentage of sperm abnormalities. Moreover, PS-NPs caused oxidative stress and triggered the p38 mitogen-activated protein kinase (MAPK) signaling pathway, as well as inhibiting the nuclear factor erythroid 2-related factor 2 (Nrf2) pathway. There was also a concomitant decrease in the expression of major proteins related to BTB, showing defected barrier integrity. A combination of these outcomes suggests that PS-NPs have the potential to interfere with the reproductive health of males, both via an oxidative and a signaling-mediated mechanism [103]. The toxicity of 39 nm polystyrene nanoplastics (PS-NPs) in male rats fed 1–10 mg/kg/day were studied over a period of five weeks. Findings demonstrated dose-related drops in testosterone, LH, and FSH concentrations, combined with reproductive tissue damage at minimal doses. The greater the exposure, the more the sperm DNA fragmentation, abnormal morphology, and low viability were observed. PS-NPs suppressed the expression of important genes in spermatogenesis, which is evidence of testicular dysfunction and HPG axis dysregulation. LH and FSH gene expression was enhanced at the maximum dose whereas ABP was non-linear. GnRH increased in all groups, yet sensitivity to GnRH dropped at increased exposures, indicating complicated endocrine-disrupting actions [104]. Toxicokinetic characteristics of microplastics (MPs) suggest that there is a high likelihood of transgenerational effects on reproduction. As technological surveys have indicated, maternal exposure to MPs during pregnancy may result in negative developmental consequences for the child in rodent models. These are a reduction in the birth weights, impairments in the homeostasis of the steroid hormones, and retarded gonadal maturation. These findings indicate that prenatal exposure to MPs can disrupt the maturation of the reproductive system and endocrine function, which could impair the reproductive health and future generations of rats [105]. Farm animals are specifically vulnerable to microplastics (MPs) because of their constant ingestion of contaminated pasture, forage, concentrate food, and water sources. The prevalence of MPs in farm soils, water bodies, and feedstuffs has sparked increased fears about how dangerous it is to be exposed chronically and the possible effects that this exposure would have on reproductive health in the long run [106]. Microplastics can also be stored in the gastrointestinal system after being ingested and later become dislodged and move to reproductive organs through systemic circulation. Lasting and long-term exposure could cause cumulative harm to the reproductive tissues, which could lead to a reduction in fertility and reproductive efficiency. The implications of such effects are of great importance to the productivity of livestock and thus the sustainability of animal agriculture economically, which is being proven in a growing number of studies. Since existing data are mainly based on manipulated laboratory models, the practical effects of persistent low-dose MNP exposure on livestock reproductive fitness itself is an open question that should be further investigated in the field.

### 2.5. Nervous System and Immunity

The neurotoxic effects of MPs and NPs on the human brain have not been studied because the research in this field is scarce. However, neurotoxicity has been found in some organisms in which the immune system of the brain caused oxidative stress in response to the particles. The reason why this damage was done could possibly be due to direct contact with the minute plastic particles or the circulation of the pro-inflammatory cytokines that led to irreversible destruction of the neurons [107]. This uncertainty is because the lack of sufficient human research data has led to researchers turning to studies on different animal models, such as zebrafish and European sea bass [83]. This is important because these disruptions support the systemic effect of micro- and nanoplastics (MPs-NPs) on immune homeostasis, and this has a major impact on the health and productivity of farm animals. In livestock and poultry, exposure to MPs-NPs has been reported to disrupt key immune functions, especially phagocytosis, which is one of the mechanisms through which the macrophage and other immune cells destroy pathogens. This impairs the phagocytic response, hence making the animals more susceptible to infections and other related illnesses [108]. As a result of the changes in the levels of neurotransmitters and disturbed movement patterns, the same species’ ability to swim was proven to have decreased. These findings were construed as a neural activity. The build-up of MPs and NPs in the fish brain tissues led to oxidative damage, which, in turn, led to the death of the cells and damaged the spatial recognition memory in the fish. In vitro tests have proved that MPs and NPs can penetrate the blood–brain barrier. The results of the studies that have been performed on the basis of animal models showed that MNPs can spread between dendritic and M cells accumulated in the body, which leads to the weakening of immunity and the ability of MPs and NPs to move through the circulatory and lymphatic systems [109]. Therefore, exposure to MPs and NPs can be tracked as a marker of neurotoxicity. Deciding on the nature of particles being dealt with, the exposure path, and the levels to which individuals have been exposed is of extreme importance. Trauma can be done through ingestion, inhalation and retrograde transmission, among others. Moreover, the level of inhalation (including the possibility of particles penetrating through the lungs or gills, nasal epithelium and blood–brain barrier and translocation of the particles to other body organs) must also be evaluated. Besides the above, it is also necessary to assess the weathering, size, shape and type of the particles. The above tests will help in clarifying each of the above pathways by which the MPs and the NPs permeate the blood–brain barrier, either by the olfactory nerve, the gustatory nerve or bot [110]. In addition, microplastics (MPs) might act as vectors of dangerous pollutants of the environment, including polycyclic aromatic hydrocarbons (PAHs), and thereby enhance immune dysregulation and reduce immune defenses. This has been highly reported in sea creatures that have been subjected to MPs contaminated with said toxicants [111]. The immunotoxic effects of MPs-NPs clearly indicate the necessity to carry out more studies on their potential long-term effects on the health of farm animals. The immunological pathway of MP-NP exposure leading to disease resistance is not only correlated but also could cause chronic inflammation, increasing the chance of developing autoimmune pathology and organ damage in animals. The existence of these adverse outcomes boosts the concern that further research and mitigation measures are required to deter the collapse of livestock and poultry industry productivity and health in the long term. When cells were treated with MPs, including polystyrene and bisphenol A, there was an increase in the concentration of lipid peroxidation (LPO) and a decrease in the release of the acetylcholinesterase (AChE) enzyme, which together led to the onset of oxidative stress [112]. Exposure to micro- and nanoplastics (MPs/NPs) has been linked to poorer swimming performance in fish, possibly due to disrupted neurotransmitter levels and abnormal movement, with behavior commonly used as a proxy for neural function. Their accumulation in brain tissue may cause oxidative damage and cell death, which can impair memory and spatial recognition [113]. A variety of metal nanoparticles (NPs), such as silver, gold, cerium oxide, aluminum oxide, titanium oxide and zirconium oxide, and other nanomaterials, including carbon-based (fullerenes and graphene), polystyrene (PS) and polyethylene (PE), were characterized in a study performed in 2017. Also, the authors examined the impact of MPs on human brain and epithelial cells, using the T98G and HeLa cell lines. The results of the research point to the fact that MPs could induce cytotoxicity in T98G and HeLa cells. Also, polypropylene (PP) particle usage demonstrated detrimental outcomes on a variety of cell populations depending on their size (20 µm and 25–200 µm) and the different quantities used in the course of each experiment. It has been proven that exposure of human beings to microplastics may lead to hypersensitivity, hemolysis and cytotoxicity, creating reasons to be concerned about the health of the general population [114].

### 2.6. Dermal Contact with Microplastics from Food Contact Materials: Pathways and Skin Health Considerations

Moreover, nanoplastics have also been found in various health and beauty products but mostly those aimed at coming into direct contact with the skin, including facial and body washes [115]. As one may observe, the above method of medication delivery by superficial application is a noteworthy method of exposure. The other significant approach of medication exposure delivered through superficial application is through the application of nanocarriers. The degree to which the skin can be penetrated is hugely dependent on the minute particle sizes and the stressed state of the skin, although there is a dearth of conclusive evidence regarding the actions of nanocarriers [116]. None of the recent research has shown any results regarding the possibility of nanoplastics penetrating the skin. The possibility of textile-engineered nanoparticles entering the epidermal barrier at low concentrations has been mentioned in just one study [117]. The absorption of MPs and NPs into the body may come about due to the consumption of objects that are contaminated with these pollutants. These products are water, cosmetics and health products. Although points of entry of plastic particles to the human body can be through sweat ducts, skin wounds or hair follicles, it is not possible that microplastics and nanoparticles (MPs and NPs) can be absorbed through the stratum corneum because of their hydrophobic nature originating from polluted sources such as water [118]. Another fact that should be mentioned is that microplastics and nanoparticles may enter the human body when scrubs are washed. The washing of scrubs is another interesting route for micro- and nanoplastics to enter a human body. Yet, particles (NPs and MPs) less than 100 nm cannot permeate the layer of the corneum. There are therefore few chances of microplastics being absorbed through the skin. Nevertheless, skin could be an entry point for nanoplastics due to the same reason. The distribution of plastic particles in the human body after their entry there and their distribution in the dermal tissue. The authors of the paper used pig skin tissue and using fluorescent polystyrene nanoparticles with the size of 20 to 200 nm [119]. The skin confocal laser scanning micrograph showed that the concentration of polystyrene nanoplastics of 20 nm diameter in the hair follicles was much higher than the concentration of polystyrene nanoplastics of 200 nm diameter in the hair follicles. However, neither particles could become ingrained in the deeper dermal tissue through penetration of the stratum corneum. These conclusions can be justified by the findings of the research done by Campbell et al., which also suggests that the outermost layers of the skin could be infiltrated only by the polystyrene particles of the diameter range of 20–200 nm [120]. The presence of cyanoacrylate follicular stripping on the perifollicular tissue of skin explants was observed to be characterized by the presence of fluorescent polystyrene nanoparticles of about 40 nm diameter. The experiment has shown that transcutaneously placed particles were later absorbed by Langerhans cells [121]. The data obtained from these earlier investigations can be employed to ascertain the probability of nanoplastics infiltrating the stratum corneum. Ultraviolet radiation-induced damage to the skin results in the weakening of the skin barrier [122]. The experiment found out that the penetration of carboxylated quantum dots into the skin was increased. An experiment conducted on the impact of UV radiation on the skin of mice showed that there was an enhancement in the capacity of carboxylated quantum dots to penetrate into the skin. Deformity of tight junction-related proteins such as zonula occludens-1, claudin-1, and occludin resulted in a change in intercellular adhesion in the study of subjects with an exposed skin [123]. Various chemical compounds, which include fatty acids, glycol, sulphoxide, surfactants, terpenes, cyclic amides, and short-chain and long-chain alcohols, are used to improve infiltration of drugs and formulations into the skin barrier [124]. The combination of urea, glycerol, and 2-hydroxyl acids/alpha hydroxyl acid is a characteristic found in body lotions. In addition, it has also been proven that the addition of these ingredients increases the ability of nanoparticles to cross the epidermal barrier [125]. In addition, the process that is used to produce the microbeads used in body and facial scrubs increases the chances that NPs may degrade into other harmful compounds which can, nevertheless, have a negative influence on human health.

### 2.7. Renal Handling of Micro- and Nanoplastics: Exposure Routes, Clearance, and Potential Kidney Effects

**Figure 4:**
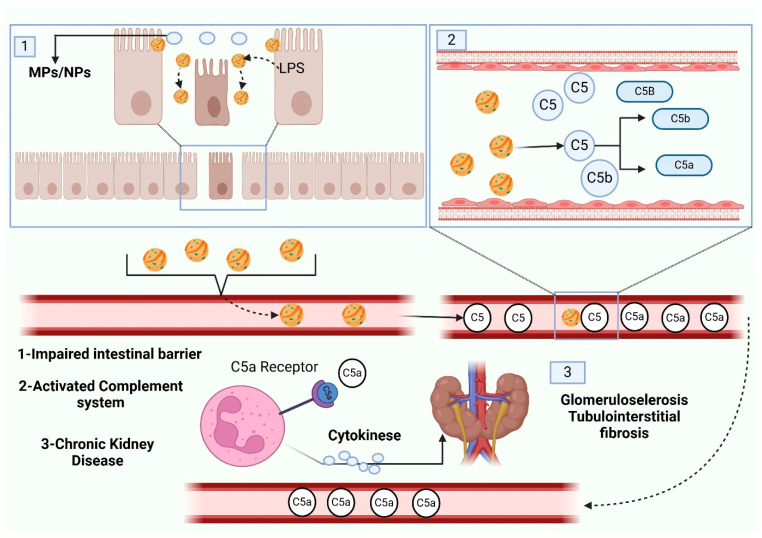
Impact of microplastics and nanoplastics on the intestinal barrier, complement system, and kidney health.

With the liver and kidneys being the primary excretion organs in the body, and given the dumping of most unwanted elements in the blood and any other secretions into them, the appearance of NPs and MPs could have a negative influence on the well-being of the nephrotic tissue, not to mention the excretory system itself. This is because of the small size and convenience of transportation of these particles. Long-term exposure to MPs and polystyrene has been proven to be a risk factor with regard to impaired kidney health. Through the work on vertebrates including mice, it has been proven that exposure of mice to MPs induced endoplasmic reticulum stress, internal inflammation, mitochondrial dysfunction, and autophagy in renal tissues [125]. Microplastics (MPs) and nanoparticles (NPs) have exhibited damaging effects on organs such as the kidneys, liver, and gut. They have the potential to develop intestinal inflammation, alter the gut microbiome, and lead to liver lipid accumulation, lipid metabolism alterations, and hepatic biomarker disturbances. Other important signaling pathways that MPs/NPs influence include AKT/mTOR and MAPK. In vitro tests show renal damage and changes in creatinine levels, and post-mortem data confirm the presence of MPs in the kidneys, spleen, and other organs. As schematically depicted in Figure 4.

The intestinal barrier is disrupted upon the intake of micro- and/or nanoplastics (MPs and NPs) into the bowel mucosa, alongside the presence of lipopolysaccharides (LPSs), which inhibits the maintenance of gut barrier integrity and, consequently, the release of particles into the bloodstream. After entering into the circulation, the MPs and NPs can trigger the complement system, specifically the C5 component leading to the generation of C5a and C5b. As a result of this immune response, cytokines are released and they help in the process of inflammation. Continuous complement system activation and subsequent inflammation may result in chronic kidney disease (CKD) such as glomerulosclerosis and tubulointerstitial fibrosis. Examples show that MPs and NPs destroy the intestinal barrier, using the complement system, and stimulate the occurrence of kidney disease. Human populations are especially susceptible to the negative impact of microplastics because of the massive consumption of seafoods exposing individuals to more risks. According to the existing literature, over 90 percent of the microplastics and nanoparticles consumed enter the environment as garbage. The shape, polymer structure, and chemicals involved in the production of MPs determine the reaction kinetics to the clearance and retention of the particles in the human body [126]. Preliminary studies have identified several adverse effects, some of which may have serious consequences. These include increased inflammatory responses, size-dependent toxicity of microplastics (MPs) that can disrupt gut bacteria and enzyme activity, and a phenomenon known as plastic particle adsorption. This process involves the mass adsorption of plastic particles onto other substances, potentially resulting in the destruction of gut bacteria. Additionally, other physiological effects of accumulated MPs have been observed, although these are less well-documented [127]. The process of microplastic absorption is influenced by several factors, including particle size, hydrophobicity, shape, surface charge, functional groups, and buoyancy [128]. Studies using mammalian models have shown that certain microplastics (MPs) can penetrate living cells, such as dendritic cells. These cells can then migrate through the lymphatic and circulatory systems, leading to the accumulation of MPs in secondary organs and potentially impacting the health of internal organs and cells [129]. Although inhaled particles may disrupt epithelial tissue involved in ingestion, microplastics (MPs) released from surgical substances and materials are primarily absorbed into tissue and the bloodstream, where they may gradually mimic the effects of endogenous enzymes or particles. Additionally, research demonstrates that bacteria present on the surface of ingested MPs can serve as vectors for pathogenic bacteria. These interactions manifest physiologically in marine animals, affecting nutritional, immunological, developmental, and toxicological processes. Ingestion of MPs has been associated with necrosis, cellular proliferation, and the formation of inflammatory tissue [54]. Research on the blue crab (Callinectes sapidus) indicates that ingestion of plastic microspheres stimulates hemocyte aggregation, subsequently impairing respiratory performance [112].

### 2.8. Inflammatory Consequences of Micro- and Nanoplastics: From Exposure to Systemic Effects

In vitro experiments reveal that bigger polystyrene particles (202 nm and 535 nm) are associated with a greater inflammatory response in HSA549 lung carcinoma cells, where more IL-8 is expressed than with smaller 64 nm particles. In the same manner, human gastric, leukemia, and lymphoma cell lines show increased IL-6 and IL-8 gene expression upon exposure to untreated and carboxylated PS nanoparticles. These results imply that it is particle size rather than surface charge that is the dominant factor leading to an inflammatory response [130]. M1-associated markers, such as CD86, NOS2, and TNFalpha, and IL1B expression did not vary when the effect of carboxylated and amino-immobilized polystyrene particles (120 nm) on the differentiation of human macrophages to either the M1 or M2 phenotype was investigated. On the contrary, exposure to either type of nanoparticle led to reduced production of IL-10 and downregulation of the scavenger receptors CD163 and CD200R in M2 cells. The phagocytic activity against Escherichia coli by M1 and M2 macrophages was reduced when amino acid-modified particles were presented. Nevertheless, the phagocytic ability of M2 macrophages to the particles itself was unaffected by carboxylated particles. Logically, in addition, carboxylated particles elevated total protein levels in both M1 and M2 macrophages, enhanced TGF-b1 release in M1 cells and also increased ATP levels in M2 cells [131].

**Figure 5:**
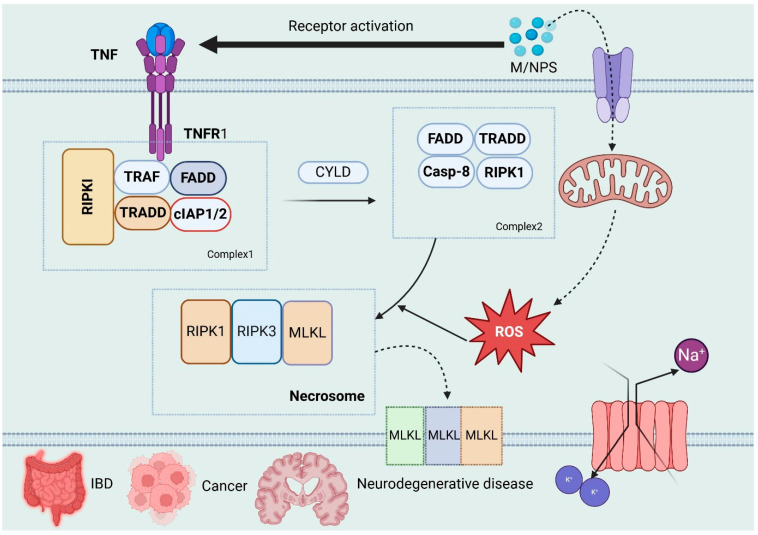
TNF signaling pathway activation by microplastics/nanoplastics and its link to disease.

An in vitro study later reported that polyethylene of 0.3–10 um diameter without any modifications caused significant cytokine production in murine macrophages and this included tumor necrosis factor-a, interleukin-1β, and interleukin-6 [132]. It has also been found that high levels of plastic particles between 0.2 and 10 um are present in the periprosthetic tissue of ultra-high-molecular-weight polyethylene implants. In addition, the presence of the macrophages in the surrounding tissue indicates an induced inflammatory response [133]. The current study has established that PS-NPs and PS-MPs induce cytotoxic and pro-inflammatory effects on macrophages, which could explain the pro-inflammatory effects of M/NPs on the gastrointestinal tract, which was previously observed in previous animal research. In addition, the impact of nanoparticles was more adverse as compared to that of microparticles. Also, our results suggest that cytotoxicity and pro-inflammatory effects caused by M/NPs can be mediated by the elevated levels of intracellular reactive oxygen species (ROS) and nitric oxide (NO), which provides information about the likely molecular mechanisms that take place [134]. Ecologically, a literature systematic review involving applications of adverse outcome pathway (AOP) principles showed that metal-based nanoparticles (MP/NPs) induce reactive oxygen species (ROS) production, therefore triggering undesirable effects (retarded development and distorted behavior). It seems that mediators of these outcomes are oxidative stress pathways and inflammatory mechanisms. The use of AOPs in the research outcomes on the public health consequences of MP/NPs proves that oxidative stress along with the pathways linked to it, such as the inflammatory reactions, can be a determining factor of critical events [135]. The impact of nanoplastics when LPS is present as a second stimulus is worthy of further investigation. LPS, a significant component of the outer cell wall of Gram-negative bacteria, has been demonstrated to strongly induce inflammation in moDCs and MOs. When lipopolysaccharide (LPS) attaches to toll-like receptor 4 (TLR4), it activates the nuclear factor (NF)-κB signaling pathway, which in turn controls the signaling of pro-inflammatory cytokines [136]. This is summarized in Figure 5.

### 2.9. Microplastic-Induced Oxidative Stress and Apoptosis: Mechanistic Pathways and Health Implications

**Figure 6:**
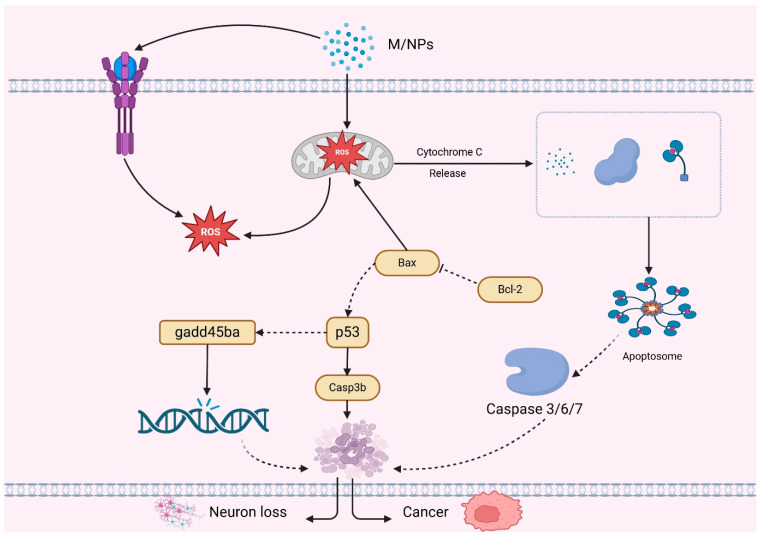
The diagrammatic illustration demonstrates the processes by which microplastics and nanoplastics induce programmed cell death and other forms of cellular damage.

There are several in vitro studies that indicate that certain polystyrene nanoparticles cause oxidative stress, apoptosis, and autophagic cell death and the responses of the particles depend on the cellular setting. Indicatively, research has established that amine-modified polystyrene nanoparticles have a high affinity to mucin, which causes the apoptosis of both the mucin-secreting intestinal epithelial cells and non-secreting ones [137]. Previous studies have been able to precipitate endoplasmic reticulum (ER) stress and the release of reactive oxygen species (ROS) within murine macrophages and pulmonary epithelial cells in response to exposure to cationic polystyrene nanoparticles. It results in the autophagic death of RAW 264.7 mouse macrophages and BEAS 2B lung epithelial cells [138]. The liver, duodenum, ileum, jejunum, large intestine, testes, lungs, heart, spleen, and kidneys of mouse subjects showed no significant negative consequences following oral exposure to a mixture of microplastics despite the cytotoxic effects that were noted in the corresponding in vitro models. Recent studies have found the stimulation of oxidative stress to be among the negative effects of polystyrene microplastics (PS-MPs). It is well known that the imbalance of the creation of reactive oxygen species (ROS) and the functionality of the antioxidant defense system are the central causative agents of oxidative stress [139]. One investigation revealed that PS-MPs significantly elevated the levels of ROS and malondialdehyde (MDA) while concurrently reducing the levels of glutathione (GSH) in the testicular tissue of rats [103]. In rats, the administration of polystyrene microplastics has been demonstrated to induce the development of fibrosis. This occurs via the stimulation of the Wnt/β-catenin signaling system. Additionally, polystyrene microplastics have been shown to cause the destruction of granulosa cells within the ovaries. This occurs through the action of oxidative stress and results in a reduction in ovarian reserve capability. The findings may offer further evidence that PS-MPs are detrimental to female reproductive health [102]. The findings of the research study indicated that the presence of polyethylene microplastics led to an enhancement in the expression of several biomarkers associated with apoptotic processes, including p53, caspase-3, caspase-9, and Bax, along with the activation of the nuclear factor-κB (NF-κB) pathway, specifically NF-κB p65, IKKα, and IKKβ. A significant component of innate immunity, nucleotide-binding oligomerization domain-like receptor protein 3 (NLRP3) inflammasomes exhibited hyperactivity. Similarly, there was a substantial decline in anti-inflammatory factors (IL-4 and IL-10) and a notable increase in pro-inflammatory factors (TNF-α, IFN-γ, IL-2, IL-6, IL-8, and IL-1β), which contribute to the development of immunological disorders. The findings indicate that oxidative stress plays a role in inflammatory immunological responses, acting as an apoptotic activation signal initiated by the NF-κB pathway and activating the NLRP3 inflammasome [133]. As illustrated in Figure 6.

### 2.10. The Potential Consequences of Exposure to Microplastics on the Metabolic Balance and Composition of Intestinal Microbiota

**Figure 7:**
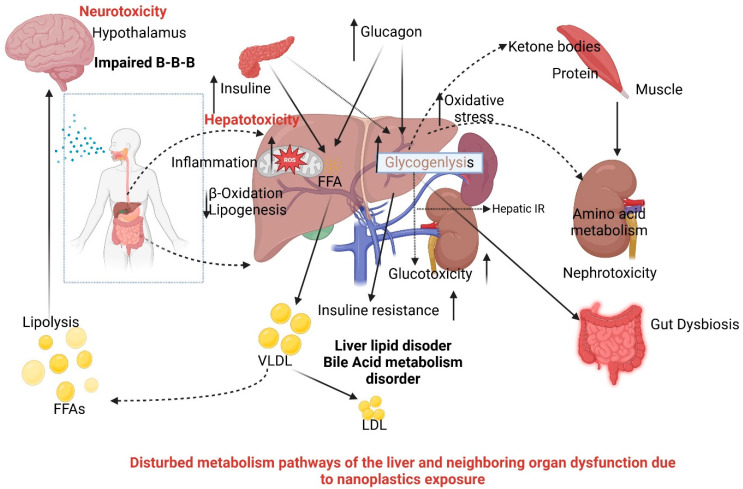
Metabolic disruptions in the liver and neighboring organs induced by nanoplastic exposure.

The recent studies have found out that microplastics and nanoplastics have the ability to regulate cellular metabolism in both in vitro and in vivo model systems, thus triggering inflammatory response conditions and the programmed cell death process. Polystyrene nanoparticles interact with the cytoplasmic membrane in order to introduce alterations in the signaling cascades in the airway epithelial cells. Empirical data reveals that human pulmonary cells express basolateral potassium ion channels that are opened when the cells are exposed to carboxylated polystyrene nanoparticles that have negative surface charge and have a nominal particle size of 20 nm [140]. Pregnant ICR mice were exposed to and treated with drinking water that was supplemented with either 0.05 mm polystyrene nanoplastics (0.05 mm PS-NPs) or 5 mm polystyrene microplastics (5 mm PS-MPs) starting right after conception and with the treatment persisting until weaning. The effects of early-life exposure to micro- and nanoplastics of various sizes on growth, development, intestinal microbiota and metabolism in young mice were determined by verifying histopathological results and performing microbiology work analyzing 16S rRNA sequences as microbiome profiles and strategic metabolomic studies. In the results, it was proven that MNPs biodistribute size-dependently, with the smaller particles accumulating more in the organs. Moreover, 0.05 m and 5.0 m PS-MNPs were permeated through the placental and mammary barriers. Further analysis showed that the bigger PS-MPs of 5 μm size showed the least accumulation in the organ [141]. After the consumption of microplastics via contaminated water and food, there is a high likelihood that there will be negative effects on gastrointestinal health. The well-being of gastrointestinal and general systemic health largely relies on the health of the gut barrier, which is inseparably related to immune activity, gut microbiota, and mucus layers, epithelium organization, and intestinal stem cells (ISCs) [142]. As schematically depicted in Figure 7.

To date, the impact of microplastics on intestinal stem cells (ISCs), key regulators of proliferation and differentiation required for gut homeostasis, remains unexplored. The effects of microplastics (MPs) in drinking water were investigated over a 42-day period in C57BL/6 mice. The mice were provided with drinking water with MPs or without MPs. No discernible differences in body weight were observed between the mice exposed to MPs and the control group. The levels of IL-1β and IL-6 gene expression in the liver and jejunum following exposure to MPs were comparable to those of the control mice. It is noteworthy that mice that consumed MPs exhibited elevated levels of IL-1β and IL-6 gene expression in their colon. Moreover, fluorescent MPs were demonstrated to be present in [143]. The aforementioned findings indicate that zebrafish display aberrant neurobehavior and impaired learning and memory when exposed to ecologically relevant concentrations of NPs and As. It is evident that disruption to the homeostasis of the microbiota–intestine–brain axis, particularly the 5-HT system, may be a potential cause of this toxicity. The disruption of intestinal health and modification of the structure of the intestinal microbiota, which can be brought about by nanoplastics, may be a contributing factor.

## 3. Conclusions and Outlook

In the agricultural system, micro- and nanoplastics (MPs/NPs) have become a topic of increasing concern, and their occurrence in livestock exposure pathways (in feed, water, soil, and air) has been demonstrated. Research has verified ingestion and translocation into animal tissues such as blood, which has implications on animal health. Mechanistically, MPs/NPs have been associated with oxidative stress, inflammation, gut barrier perturbation, immune dysregulation and endocrine action, which may adversely affect animal productivity and animal welfare. Moreover, animal-related products such as milk can be contaminated during various steps of farm processing, usually by wear and tear of equipment and filtration systems. Since MPs/NPs have the ability to support toxic chemical additives, they are direct and indirect food safety hazards. To resolve this issue, it is necessary to take an integrated One Health approach that involves the intervention of environmental, veterinary and food systems. Harmonized detection methods (particularly in nanoplastics), realistic exposure assessment in farm animals, and process-aware mitigation (lessen plastic imports and wastes in agriculture, have greater control over feed and water quality, reduce particle shedding on farm and processing equipment, and create safer-by-design materials in food-contact uses) are among the priorities. To achieve transparency and scientific rigor, this review differentiates between detection-based evidence and toxicological findings, critically describes the hierarchy of evidence among the types of studies, and recognizes the high degree of heterogeneity of experimental designs. These considerations are needed to put the results in context and to inform future studies on environmentally pertinent and biologically significant exposure scenarios.

## Figures and Tables

**Table 1:** An overview of the potential effects of MNPs on various organ systems in animals and humans using a One Health approach.

**Animal**	**Types of Microplastics**	**Amount Isolated**	**Organs Isolated From**	**Size of Microplastics**	**Reference**
Cow	Polyvinyl chloride (PVC), polyethylene (PE), polystyrene-based polymers, and polypropylene (PP)	Polyvinyl chloride particles (PVC-Ps) have been reported to range between 1.2 and 6.1 µg/g, while polystyrene-based particles (Styr-Ps) vary from 0.09 to 1.5 µg/g. Polyethylene (PE) concentrations have been observed within a range of 0.22 to 2.9 µg/g.	Blood	≥700 ns	[62]
Cow	Nylon (a synthetic polyamide polymer)	An average concentration of 0.14 particles per gram.	Muscle tissue (meat), liver, and gastrointestinal organs (tripe)	Less than 5 mm in size	[67]
Sheep	Fiber	0.13 items/g.	Meat, liver, and tripe	<5 mm	[67]
Pig	Styrene-based polymers, including polystyrene and its derivatives	Polyvinyl chloride particles (PVC-Ps) have been reported to range from 1.7 to 17 µg/g, while styrene-based polymers (Styr-Ps) range from 0.3 to 10 µg/g. Polyethylene (PE) concentrations are observed to be higher, ranging from 2.1 to 33 µg/g.	Blood	Equal to or greater than 700 ns in size	[62]
Chicken	Not specified	Detected.	Eggs	Not specified	[66]
Chicken	Polyvinyl chloride (PVC), low-density polyethylene (LDPE), polystyrene (PS), and polypropylene homopolymer (PPH)	An average of 17.8 ± 12.1 particles per sample in the crop and 33.25 ± 17.8 particles per sample in the gizzard, indicating a higher accumulation of particles within the digestive organs.	Crop and gizzard	50–500 µm	[35]
**Name**	**Species**	**Research Design**	**Granulation/Exposure**	**Bioactivity**	**Reference**
**GIT**	Human	The in vitro design encompasses human colon adenocarcinoma Caco-2 cells and colon adenocarcinoma HT29-MTX cells.	Cells were exposed to polyethylene microplastics (PE-MPs) with a diameter of 1–10 μm for 14 days. The daily exposure was conducted in a manner whereby 21 mg of PE-MPs were suspended in 8 mL of water, containing 0.01% (*w*/*v*).	A rise in the number of detrimental microbes, including those belonging to the Enterobacteriaceae, Desulfovibrionaceae and Pathobiont families, was observed. Concurrently, there was a decline in the population of beneficial bacteria, namely those belonging to the Christensenellaceae family.	[68]
	Human	The stimulation of the gastrointestinal (GIT) tract was achieved by combining a harmonized static model with a dynamic gastrointestinal simulation model in an in vitro setting.	The administration of a single dose of digested polystyrene microplastics (0.166 g) over a period of 72 h, referred to as PET-MPs.	Biotransformation of PET-MPs in the gastrointestinal tract (GIT) and colon. The structure of PET-MPs was observed to differ from that of the original particle.	[69]
	Mice	The study employed C57BL/6 J mice and an in vivo experimental design.	A combination of PS-NPs (50 nm and 500 nm, respectively) and PS-MPs (administered orally by gavage at a dose of 20 mL/kg bw) was given on a daily basis for a period of 28 days in combination with PS MNPs (administered orally by gavage at a volume of 20 mL/kg bw).	Intestinal toxicity: Impairment of the intestinal barrier function; Reactive oxygen species (ROS)-mediated apoptosis of epithelial cells.	[70]
**Respiratory system**	Human	The in vitro engineering of human bronchial epithelial cells (BEAS-2B) represents a pivotal aspect of biomedical research.	Postscript: The cells were treated with the MPs at concentrations ranging from 1 to 1000 μg/cm^2^ for 24 and 48 h.	Cytotoxic and inflammatory effects via ROS production: May result in a reduction in the transepithelial electrical resistance; May increase the risk of COPD.	[71]
	Rat	This study employs a Sprague–Dawley rat model in vivo design.	A series of PS-MPs with dimensions of 100 nm, 500 nm, 1 μm, and 2.5 μm were administered via inhalation for a period of three days. Following this, an intra-tracheal instillation of either saline or the aforementioned PS-MPs at concentrations of 0, 0.5, 1, and 2 mg per 200 μL was conducted for a period of two weeks.	Histological alterations were observed in the lungs as a consequence of the accumulation of particles in the size range of 100 nm to 1 μm, characterized as polyvinyl sulfate-modified microparticles (PS-MPs). Additionally, an increase in the expression of pro-inflammatory cytokines, including IL-6, TNF-α, and IL-1β, was documented. It can be postulated that these changes may contribute to the development of lung inflammation.	[72]
**CVS**	Human	The in vitro design of human embryos and human-induced pluripotent stem cells (hiPSCs)	PS-NPs (40 nm and 200 nm), derived from hiPSCs, were subjected to 24 h of exposure at a concentration of 1 × 10^9^/mL of 40 nm PS-NPs.	The expression of the LEFTY1 and LEFTY2 genes was reduced, while the expression of the CA4 and OCLM genes was elevated. These changes affected the development of the atrioventricular heart valve.	[73]
	Human	The objective of this study is to develop an in vitro design for red blood cell (RBC) production.	Precipitated silica nanoparticles (PS-NPs), measuring 50–250 nm, were incubated at concentrations ranging from 50 to 500 μg/mL for an hour.	The phenomenon of induced hemolysis was observed in a size- and dose-dependent manner in a plasma-free medium. However, this phenomenon was not observed in a full plasma environment.	[74]
	Chicken	In vivo design of chicks at 61 days of age.	P.S.—MPs (5 μm) were orally administered in doses of 1–100 mg/L for a period of six weeks.	The pathological damage and ultrastructural changes observed in the heart are the result of two main processes: induced oxidative stress and induced myocardial pyroptosis and cellular inflammation.	[75]
**Hepatic system**	Human	Hepatocellular (Hep G2) liver cells are used as a model for studying liver function.	Hep G2 cells were treated with 5 μg/mL of green fluorescent PS-MPs (1 μm) for 24, 48 and 72 h.	A reduction in cell viability was observed, accompanied by morphological alterations in cells that had taken up MPs. Additionally, there was a notable decline in glycolytic activity.	[76]
	Mice	The study comprised two experimental groups, namely a control group and an exposed group, which were maintained under identical conditions in vivo.	The fluorescent and pristine PS-MPs were treated with a dosage of 0.01 to 0.5 mg/day via oral gavage for a period of up to 28 days. The PS-MPs were 5 and 20 μm in size, respectively.	The accumulation of MPs in liver tissue was observed to result in the following disturbances: A reduction in ATP levels, which disrupts energy metabolism; A reduction in TC and TG levels, which disrupts lipid metabolism.	[77]
**Renal system**	Human	The in vitro cell culture design utilizes embryonic kidney (HEK 293) cells.	Green fluorescent PS-MPs (1 μm) were used, and HEK 293 cells underwent a 24-, 48-, and 72 h treatment regimen with 5 μg/mL (100 μg/mL PS for cell proliferation assay) PS-MPs.	An increase in cellular reactive oxygen species (ROS) production was observed. A decrease in glycolytic activity was observed.	[78]
	Mice	The male mice were divided into five groups for the in vivo degradation study.	The study involved the exposure of polystyrene nanoparticles (PS-NPs) with a diameter of 50 nm and polystyrene micro-particles (PS-MPs) with a diameter of 300 nm, 600 nm and 4 μm to a solution of 5 mg of water over a period of 24 h and four weeks, respectively.	The process of bioaccumulation, coupled with the exacerbated biotoxicity of the substance in question, resulted in notable alterations to the subject’s histomorphology. Additionally, there was a notable decline in weight, an elevated mortality rate, and a shift in several biomarkers.	[79]
**Reproduction and development**	Human	The study involved 18 mother–infant pairs from Shanghai, China, and was conducted as a prospective pilot study in 2021.	A total of eighteen placental samples and twelve meconium samples were collected for the purpose of detecting the presence of microplastic particles (MPPs).	MPs were identified in 76.5% of the samples. Sixteen different types of MPs were observed, with polyamide and polyurethane representing the most prevalent. The predominant microbiota included Proteobacteria, Bacteroidota, and Firmicutes, with notable differences in β-diversity.	[55]
	Mice	The study employed an in vivo design, with female mice as the subject population.	The experimental procedure involved the administration of PS-MPs/G-PS-MPs (0.79 µm, administered orally via gastric gavage at a dose of 30 mg/kg over a period of 35 days).	An accumulation of PS-MPs was observed in a number of organs, including the uterus, ovary and blood vessels. In addition, there was a reduction in the levels of reduced glutathione (GSH) and matrix metalloproteinases (MMP) and ROS.	[80]
**CNS**	Human	The presented model of the human forebrain is three-dimensional and comprises cortical spheroids that were designed in vitro.	Polyvinylsiloxane (PVS) particles of 1 and 10 microns in diameter were subjected to exposure at concentrations of 100, 50, and 5 micrograms per milliliter during the period between days four and ten and days four and thirty.	The results demonstrated a reduction in cell viability and a downregulation of mature neuronal markers and cortical layer VI markers in response to long-term exposure to PS-MPs. Furthermore, the observed effects were found to be dose-dependent, exhibiting a correlation with particle size.	[81]

## Data Availability

No new data were created or analyzed in this study.
